# *In Vitro* Cytotoxic Potential of Essential Oils of *Eucalyptus benthamii* and Its Related Terpenes on Tumor Cell Lines

**DOI:** 10.1155/2012/342652

**Published:** 2012-05-08

**Authors:** Patrícia Mathias Döll-Boscardin, Adilson Sartoratto, Beatriz Helena Lameiro de Noronha Sales Maia, Josiane Padilha de Paula, Tomoe Nakashima, Paulo Vitor Farago, Carla Cristine Kanunfre

**Affiliations:** ^1^Postgraduate Program in Pharmaceutical Sciences, Federal University of Paraná, 632 Prefeito Lothário Meissner Avenida, 80210-170 Curitiba, PR, Brazil; ^2^Research Center for Chemistry, Biology and Agriculture, University of Campinas, P.O. Box 6171, 13081-970 Campinas, SP, Brazil; ^3^Department of Chemistry, Federal University of Paraná, Polytechnic Center, P.O. Box 19081, 81531-990 Curitiba, PR, Brazil; ^4^Postgraduate Program in Pharmaceutical Sciences, State University of Ponta Grossa, 4748 Carlos Cavalcanti Avenida, 84030-900 Ponta Grossa, PR, Brazil; ^5^Department of General Biology, State University of Ponta Grossa, 4748 Carlos Cavalcanti Avenida, 84030-900 Ponta Grossa, PR, Brazil

## Abstract

*Eucalyptus* L. is traditionally used for many medicinal purposes. In particular, some *Eucalyptus* species have currently shown cytotoxic properties. Local Brazilian communities have used leaves of *E. benthamii* as a herbal remedy for various diseases, including cancer. Considering the lack of available data for supporting this cytotoxic effect, the goal of this paper was to study the *in vitro* cytotoxic potential of the essential oils from young and adult leaves of *E. benthamii* and some related terpenes (*α*-pinene, terpinen-4-ol, and *γ*-terpinene) on Jurkat, J774A.1 and HeLa cells lines. Regarding the cytotoxic activity based on MTT assay, the essential oils showed improved results than *α*-pinene and *γ*-terpinene, particularly for Jurkat and HeLa cell lines. Terpinen-4-ol revealed a cytotoxic effect against Jurkat cells similar to that observed for volatile oils. The results of LDH activity indicated that cytotoxic activity of samples against Jurkat cells probably involved cell death by apoptosis. The decrease of cell DNA content was demonstrated due to inhibition of Jurkat cells proliferation by samples as a result of cytotoxicity. In general, the essential oils from young and adult leaves of *E. benthamii* presented cytotoxicity against the investigated tumor cell lines which confirms their antitumor potential.

## 1. Introduction

*Eucalyptus* L. is a large genus of the Myrtaceae family that includes some 900 species and subspecies [1]. These evergreen tall trees are native to Australia and show a worldwide distribution. For over 60,000 years ago, Australian aborigines developed a sophisticated empirical understanding of indigenous plants such as *Eucalyptus*. They traditionally used *Eucalyptus* leaves to heal wounds and fungal infections [2]. Although their pharmacological or toxicological properties have not been thoroughly investigated, infusions and decoctions of *Eucalyptus* plants are widely used in the treatment of respiratory diseases, for example, common cold, influenza, and sinus congestion [3, 4]. In Africa, the powder of barks has been indicated as insecticide. The leaves of *E*. *globulus* and *E*. *robusta* have been recommended for treating dysentery, articular pain, and tonsillitis in China. Besides their uses in folk medicine, many studies demonstrated analgesic, expectorant, anti-inflammatory, and antimicrobial properties from leaves of *Eucalyptus* spp. [3, 4]. 

Currently, extracts and components isolated from some *Eucalyptus* species have shown to possess cytotoxic activities. Cladocalol, a formylated triterpene, was isolated from leaves of *E. cladocalyx* and showed cytotoxic effect on the myeloid leukemia cell line HL-60 [5]. A phlorogrucinol-monoterpene derivative, euglobal-G1, obtained from leaves of *E. grandis* exhibited a remarkable inhibitory effect on two-stage carcinogenesis test of mouse skin tumors induced by 7,12-dimethylbenz[**α**]anthracene [6]. Three new phenol glycosides acylated with (+)-oleuropeic acid, cypellocarpins A, B, and C, along with seven known compounds, isolated from leaves of *E. cypellocarpa* suppressed an *in vivo* two-stage carcinogenesis induced with nitric oxide and 12-O-tetradecanoyl-phorbol-13-acetate on mouse skin [7]. Ashour [8] verified that essential oils from stems of *E*. *torquata* and leaves of *E*. *sideroxylon* showed cytotoxic activities on MCF7 cells. Al-Fatimi et al. [9] investigated 14 plant species used as traditional medicine in Yemen for cytotoxic activity against human ECV-304 cells and reported that *E*. *camaldulensis* had a remarkable biological activity. These studies increase the interest in investigating the cytotoxic effect against tumor cells from other species of *Eucalyptus* with the purpose of improving the therapeutic opportunities against cancer. 

*Eucalyptus benthamii* Maiden *et* Cambage is a tall attractive smooth white-barked tree, commonly known as camden white gum or Nepean River gum. In Australia, it is listed as a vulnerable species [10]. This species was recently introduced in Southern Brazil as renewable source of timber due to fast growing and high resistance to cold [11]. Regarding the well-known medicinal properties of *Eucalyptus* species, local communities from Campos Gerais region of Paraná in Southern Brazil have used *E. benthamii* as an herbal remedy for many therapeutic purposes, for example, microbial infections and asthma. In spite of these uses, young and adult leaves of *E. benthamii* have been currently indicated as a folk practice for treating cancer [12]. In addition, this species has been taken as tea obtained through infusing its leaves in hot water or used as steam inhalations. Infusion is particularly recommended for throat, esophageal, and stomach cancers as well as lymphoma and cervical cancer. For lung cancer, family farmers and their communities are deeply inhaling the fumes resulting from the essential oil of *E. benthamii* [12]. However, medicinal investigations about the essential oil of *E. benthamii* are even lacking particularly related to its possible cytotoxic properties. A recent paper reported that the essential oil of *E. benthamii* provided larvicidal and adulticidal activities against *Aedes aegypti* [13].

Considering this lack of available data for supporting the cytotoxicity of the essential oil of *E. benthamii*, the goal of this work was to investigate the *in vitro* cytotoxic activity of the essential oil from young and adult leaves of *E. benthamii* and some related terpene compounds, **α**-pinene, terpinen-4-ol, and **γ**-terpinene on Jurkat (T leukemia cells), J774A.1 (murine macrophage tumor), and HeLa (cervical cancer) cells lines.

## 2. Materials and Methods

### 2.1. Plant Material

Young and adult leaves of *E. benthamii* were collected at Fazenda de Transferência de Tecnologia of Embrapa Florestas (altitude: 926 m, latitude: 25° 10′ 09′′ S and longitude: 50° 03′ 44′′ W) in Ponta Grossa, PR, Brazil, during the summer of 2010. The species was identified by the vouchers 59440 and 350231 and stored at the herbaria from the Biological Sciences Center in the Federal University of Paraná and Municipal Botanical Museum, respectively.

### 2.2. Extraction of Essential Oil and GC-MS Analysis

In separate, young and adult leaves of *E. benthamii* were air dried and then distilled using a Clevenger type apparatus for 6 h. The essential oils were dried with anhydrous sodium sulphate and stored in glass vial with Teflon-sealed caps at 4 ± 0.5°C in the absence of light until used. The identification of volatile constituents was performed using a Hewlett-Packard 6890 gas chromatography, equipped with a Hewlett-Packard 5975 mass selective detector and capillary column HP-5 (30 m × 0.25 mm × 0.25 *μ*m). GC-MS was carried out using split/splitless injection, with injector set at 220°C, column set at 60°C, with heating ramp of 3°C/min and final temperature at 240°C, and the detector was set at 250°C. Helium was used as carrier gas at 1 mL/min. The GC-MS electron ionization system was set at 70 eV. Quantitative analysis was carried out using a Hewlett-Packard 5890 gas chromatography equipped with a flame ionization detector under the same conditions previously described. A sample of each essential oil was dissolved in ethyl acetate (20 mg/mL) for the analyses. Retention indices (RI) were determined by injection of hydrocarbons standards and essential oil sample in the same conditions. The oils components were identified by comparison with data from literature [14] and the profiles from the mass spectra libraries (Wiley 139, 275, and 7 and Nist 127). The GC-FID quantification was obtained using GC-FID chromatogram and was expressed as mean from three samples of each extracted essential oil.

### 2.3. Samples for Cell Culture Tests

The previously obtained essential oils from young and adult leaves of *E. benthamii* and its related terpenes: (+)-**α**-pinene, (−)-terpinen-4-ol and **γ**-terpinene were used for cell culture protocols. These isolated compounds were purchased from Sigma as analytical standard grade. A stock solution (100 mg/mL) of each sample was prepared with propylene glycol and ethyl alcohol (1 : 4) as solubilizing procedure [15]. Prior to the cell experiments, these samples were diluted to final concentrations of 3, 10, 30, 100, and 300 *μ*g/mL [16, 17] using culture medium.

### 2.4. Cells and Cell Cultures

Jurkat (T leukemia cells), J774A.1 (murine macrophage tumor), and HeLa (cervical cancer) cells lines were obtained from American Type Culture Collection. All cultures were maintained in a color-free medium composed of RPMI-1640 Medium (Sigma). This medium was supplemented with 10% fetal bovine serum (FBS, Life Technologies) and containing 0.1% of antibiotic mix: 10,000 units penicillin and 10 mg streptomycin per mL (Sigma). Sodium bicarbonate (2 mg/mL) was also added. Cultures were maintained at 37°C in a humidified 5% CO_2_ incubator. Experiments were performed at concentrations of 250,000–500,000 cells per mL, and cells were in exponential growth phase at the time of testing. These cells were subcultured every 3-4 days. The viability of the cells exceeded 95% as determined by the trypan blue (0.4% trypan blue solution, Sigma) dye exclusion method.

### 2.5. *In Vitro* Cytotoxicity Tests

#### 2.5.1. MTT Assay

The cytotoxicity was carried out by MTT [3-(4,5-dimethylthiazol-2-yl)-2,5-diphenyltetrazolium bromide] (Sigma) assay for investigating changes in mitochondrial/non-mitochondrial dehydrogenase activity [18]. In brief, cells (Jurkat, J774A.1 or HeLa cells lines, 5 × 10^3^ cells/mL) were seeded on 96-well plates and cultured in RPMI 1640 containing 10% FBS at 37°C and 5% CO_2_ for 24 h. Each sample at various concentrations (3, 10, 30, 100, and 300 *μ*g/mL) was then added. Exposure periods of 24 and 72 h were chosen for determining the *in vitro* cytotoxicity. After incubation, the supernatant was removed, and MTT solution (0.5 mg/mL) was also added to each well 30 min prior to the end of the experiment. Water-insoluble dark blue formazan crystals formed in viable cells were solubilized in DMSO, and the absorbance was measured at 550 nm using a microplate reader (Biotek *μ*Quant). Cell survival was determined by comparing the absorbance values obtained for treated and untreated cells. The cytotoxicity was expressed as the concentration of sample that inhibited 50% of cell growth (IC_50_) and was calculated by Probit regression.

#### 2.5.2. Lactate Dehydrogenase (LDH) Activity Assay

In order to evaluate the activity of the cytoplasmic enzyme lactate dehydrogenase (LDH) released from the cytosol when cells were damaged or under stress, Jurkat cells (10^6^ cells) were plated into *Eppendorf* tubes. The LDH activity was determined using a commercial kit (LDH UV-PP kit, Analisa). Each sample at 300 *μ*g/mL concentration was added. After 4 h, enzymatic measurements of LDH released into the supernatant were spectrophotometrically performed at 340 nm [19]. Absorbance values were then correlated with the number of viable cells to predict the cytotoxic activity. Serum-free culture medium and 1% TritonX-100 (Sigma) were used as negative and positive controls, respectively.

#### 2.5.3. Analysis of Cell DNA Content

The effect of samples on cell proliferation activity was determined by measuring DNA content. Jurkat cells were seeded (1.5 × 10^5^ cells/mL) on 24-well plates [20]. After 24 h, each sample at 300 *μ*g/mL concentration was added, and plates were maintained at 37°C in a humidified 5% CO_2_ incubator for 48 h. A diphenylamine solution (1.5% in acetic acid) was then added, and plates were kept in dark for 24 h. The absorbance was spectrophotometrically determined at 575 nm. Cytotoxicity was expressed in percentage of DNA content. Culture medium (RPMI 1640 containing 10% FBS) and vincristine (40 nM or 36,92 ng/mL, Zodiac Pharmaceutical Products) were used as negative and positive controls, respectively.

### 2.6. Statistical Analyses

Results were evaluated by Prism software. Statistical analyses were carried out by one way ANOVA (Graph Pad Prism 5.01 Software) followed by Tukey *post hoc *test.

## 3. Results and Discussion

The chemical composition of the essential oils from young and adult leaves of *E. benthamii* is presented in Table 1. Both volatile oils consisted of a complex mixture of monoterpenes and sesquiterpenes. The main identified compounds for the essential oil from young leaves (YLEO) of *E. benthamii* were **α*-*pinene (36.82%), globulol (20.54%), aromadendrene (15.94%), and **γ**-terpinene (5.51%). The oil extracted from adult leaves (ALEO) was composed mainly by **α**-pinene (36.92%), globulol (20.22%), aromadendrene (12.40%), and **γ**-terpinene (4.38%). Therefore, the volatile compositions were quite similar regarding these main compounds. However, some differences on quantitative composition of these essential oils were particularly related to their minor compounds which can be explained by genotype conditions. It has been extensively reported that many *Eucalyptus* species show heteroblasty, producing juvenile and adult leaves differing markedly in morphology and anatomy [21]. Consequently, differences on the biosynthetic pathway can also occur and lead to a broad range of representative reaction types of terpenoid metabolism [22] which influence the final volatile composition.

Some previous papers were devoted to study the volatile chemical composition of essential oils provided by *E. benthamii*. Tian et al. [23] verified that **α**-pinene (31.00%), globulol (15.34%), aromadendrene (13.80%), and epiglobulol (4.86%) were the principal constituents of the essential oil extracted from leaves of *E. benthamii* by steam distillation. Silva et al. [24] investigated the percentage of **α**-pinene provided by the essential oil of *E. benthamii* during the seasons and observed values varying from 24.2% (spring) to 47.6% (fall). Mossi et al. [25] aimed at evaluating the insecticidal and repellency effect of five essential oils of *Eucalyptus* against *Sitophilus zeamais* Motschulsky (Coleoptera, Curculionidae) and reported that the volatile oil of *E. benthamii* contained **α**-pinene (54.04%), viridiflorol (17.12%), 1,8-cineole (9.93%), aromadendrene (7.3%), and globulol (3.61%). Lucia et al. [13] studied the fumigant and larvicidal activity of some essential oils of *Eucalyptus* against *Aedes aegypti* (Diptera, Culicidae) and showed that the essential oil of *E. benthamii* var. *benthamii* exhibited a higher content of **α**-pinene (73.15%) while the essential oil of *E. benthamii* var. *dorrigoensis* revealed 1,8-cineole (74.73%) as the major component. Therefore, the volatile chemical compositions reported in the present paper for the studied essential oils from young and adult leaves of *E. benthamii* are in accordance to the literature due to their relatively high concentrations of **α**-pinene. The differences in chemical composition can be related to soil and climate conditions, water stress, collection place, nutrition, and other abiotic factors. Moreover, the presence of subspecies and chemotypes can lead to changes in the final volatile chemical composition of the essential oil of *E*. *bentamii*. Thus the evidence of these qualitative and quantitate differences reinforces the need for establishing the chemical profile of this essential oil prior to a biological assay.

Furthermore, *E. benthamii* also revealed some particular differences in the chemical composition as compared to usual *Eucalyptus* species, since of its essential oils contained only traces of 1,8-cineole. However, many other species of *Eucalyptus* which do not contain 1,8-cineole as the major volatile compound have been revealed some remarkable pharmacological activities. In that sense, Elaissi et al. [26] screened the antibacterial activities of twenty essential oils of *Eucalyptus* species. The volatile oil of *E*. *odorata*, that showed less than 5% of 1,8-cineole, demonstrated the best inhibition zone diameter against *S*. *aureus*. Although 1,8-cineole is usually related to the treatment of respiratory diseases, other volatile components provided by some essential oils from *Eucalyptus* as camphene, globulol, limonene, **α**-pinene, **β**-pinene, and *p*-cymene have been provided some properties as antitussives and expectorants [27]. Therefore the fact that many of the therapeutic effects of the essential oils from *Eucalyptus* spp. that have been attributed to 1,8-cineole do not determine that other species that contain 1,8-cineole in trace amounts have not been used as herbal medicine.

The results for MTT assay are summarized in Tables 2 and 3 considering exposure periods of 24 and 72 h, respectively. In general, the volatile oils from young and adult leaves of *E. benthamii* showed some degree of cytotoxicity against the studied cells. Regarding the essential oil provided by young leaves (YLEO) of *E. benthamii*, Jurkat cells revealed a more sensitive response (IC_50_ = 108.33 *μ*g/mL at 24 h and IC_50_ = 56.51 *μ*g/mL at 72 h) when compared to J77A.1 cells (IC_50_ = 287.98 *μ*g/mL at 24 h and IC_50_ = 166.87 *μ*g/mL at 72 h). The essential oil obtained from adult leaves (ALEO) of *E. benthamii* demonstrated the same behavior for these two cell lines (Tables 2 and 3). Similar data were also observed for **α**-pinene, **γ**-terpinene, and terpinen-4-ol in which Jurkat cells had a more sensitive response to these compounds (IC_50_ = 192.42, 136.60, and 50.20 *μ*g/mL at 24 h and IC_50_ = 186.09, 156.92, and 54.84 *μ*g/mL at 72 h, resp.) than J774A.1. The terpenes **α**-pinene and **γ**-terpinene showed no activity against J77A.1, while terpinen-4-ol revealed 220.02 and 189.70 *μ*g/mL as IC_50_ value at 24 and 72 h, respectively. Regarding the cytotoxicity against HeLa cells, the volatile oils from young and adult leaves of *E. benthamii* showed IC_50_ of 84.24 and 110.02 *μ*g/mL at 24 h and 120.57 and 101.90 *μ*g/mL at 72 h, respectively. It was verified that **α**-pinene, **γ**-terpinene and terpinen-4-ol did not exhibit effect on this tumor cells. As proposed by previous studies [16] that performed the cytotoxic effect of essential oils, IC_50_ values between 10–50 *μ*g/mL represent a strong cytotoxic activity. Moreover, IC_50_ values between 50–100, 100-200, and 200-300 *μ*g/mL indicate moderate, weak, and very weak cytotoxic properties, respectively. Furthermore IC_50_ values higher than 300 *μ*g/mL represent no cytotoxicity.

Considering the cytotoxic activity on the three studied tumor cells based on MTT assay, the essential oils demonstrated enhanced results than **α**-pinene and **γ**-terpinene, particularly for Jurkat and HeLa cell lines. These values can be attributed to a synergic effect among monoterpenes and sesquiterpenes provided by the volatile oils. In that sense, for biological purposes, synergism appears to be more meaningful than its isolated compounds due to the activity of the main components can be modulated by other minor molecules which can lead to better cellular distribution of the essential oil [28].

MTT reduction is usually performed to study mitochondrial/nonmitochondrial dehydrogenase activity as a cytotoxic test for a variety of chemical compounds. Therefore, volatile oils from young and adult leaves of *E. benthamii* are potentially effective to change the enzymatic activity of mitochondria and initiate a preliminary injury that leads to cell death. Furthermore, it was also reported that essential oils can cause damage in the mitochondrial membrane since they provoke depolarization of the mitochondrial membranes by decreasing the membrane potential [29–31] and also alter the fluidity of membranes which become abnormally permeable. These additional mechanisms reported to essential oils can also had contributed to the cytotoxic effect of volatile oils from young and adult leaves of *E. benthamii*.

Essential oils and their individual volatile components have been brought the attention of research groups on cancer. A number of articles are devoted to investigate their effect against a variety of human cancer cell lines. De Sousa et al. [32] verified that the essential oil of lemon balm (*Melissa officinalis* L.) showed a cytotoxic activity against some human cancer cell lines (A549, MCF-7, Caco-2, HL-60, and K562) and a mouse cell line (B16F10). Regarding the cytotoxic effect of essential oils of *Eucalyptus*, data are remarkable restricted. Ashour [8] showed cytotoxic activities of volatile oils and extracts from stems, leaves, and flowers of *E*. *sideroxylon* and *E*. *torquata* against the human breast adenocarcinoma cell line (MCF7). The essential oil extracted from stems of *E*. torquata exhibited cytotoxicity against MCF7 cells followed by volatile oils from leaves of *E*. *torquata* and leaves of *E*. *sideroxylon*. 

Although the studied essential oils showed cytotoxic results against tumor cell lines, its major component **α**-pinene did not demonstrate the same behavior with only a weak response as an isolated cytotoxic agent against Jurkat cells. This result is a further evidence that the combination of volatile components of essential oils can influence the final cytotoxic effect. No cytotoxicity was observed when **α**-pinene was evaluated against J774A.1 and HeLa. This monoterpene is widely related to antibacterial and insecticide activities and can be used for industrial purposes in camphor synthesis and perfumery products [33, 34]. The monoterpene **α**-pinene has been exhibited *in vitro* cytotoxicity on HEPG2 human hepatocellular carcinoma cells [35]. The *in vitro* cytotoxicity of the essential oil and major constituents of *Cymbopogon jwarancusa* (Jones) Schult. demonstrated a percentage of inhibition less than 20% of THP-1 (human acute monocytic leukemia), A-549 (adenocarcinomic alveolar basal epithelial), HEP-2 (human liver tumor), and IGR-OV-1 (ovarian carcinoma) cell lines by **α*-*pinene (100 *μ*g/mL) [36]. Another study reported that **α*-*pinene isolated from *Schinus terebinthifolius* Raddi induced apoptosis and conferred antimetastatic protection in a melanoma model. It has been shown that **α**-pinene, while inactive alone against C32 (human amelanotic melanoma) and ACHN (human renal cell adenocarcinoma) cells, can act in synergy with other antiproliferative components of essential oils [37]. Zhou et al. [38] clarified that **α*-*pinene inhibits the nuclear translocation of NF-*κ*B which regulates the expression of genes that play critical roles in apoptosis and immunomodulation. In spite of many papers about **α*-*pinene and its cytotoxicity against tumor cells, none of them was related to Jurkat, HeLa, and J774A.1 cell lines.

The monoterpene hydrocarbon **γ**-terpinene presented some cytotoxic properties against Jurkat cell line. However, it was not observed any cytotoxicity for this volatile compound against J774A.1 and HeLa cell lines below 300 *μ*g/mL. There are few studies linking **γ**-terpinene and cytotoxicity activity. It was verified cytotoxic effects for leukemia HL-60 and NB4 cells using the essential oil obtained from dried leaves of *Majorana hortensis* which showed a content of 15.0% **γ**-terpinene [39]. Bourgou et al. [40] studied the cytotoxic activity of **γ**-terpinene against human lung carcinoma A-549 and colon adenocarcinoma DLD-1 cells and achieved IC_50_≥ 100 *μ*M (13.62 *μ*g/mL) for both cells lines.

The isolated terpinen-4-ol showed a cytotoxic effect against Jurkat cells similar to the evaluated essential oils. This monoterpene has been extensively related to antiviral, antibacterial, antifungal, and insecticidal effects as well as it has been shown antioxidant and anti-inflammatory activities [41–45]. This compound also exhibited antiproliferative and cytotoxic effects on murine AE17 mesothelioma and B16 melanoma tumor cell lines [46]. The essential oil of *Melaleuca alternifolia* and its main component, terpinen-4-ol, were able to impair the growth of human M14 melanoma cells [47]. Wu et al. [48] verified that terpinen-4-ol elicited a dose-dependent cytotoxic effect on human nonsmall cell lung cancer. Cytotoxicity of Australian tea tree oil (*M*. *alternifolia*), terpinen-4-ol, 1,8-cineole, and **α**-terpineol were investigated on five different human cell lines: HEPG2, HeLa, MOLT-4 (human acute lymphoblastic leukemia), K-562 (Human erythromyeloblastoid leukemia), and CTVR-1 (B cell-derived from bone marrow of a patient with acute myeloid leukaemia). The overall rating for cytotoxicity of tea tree oil and its components was **α**-terpineol > tea tree oil > terpinen-4-ol > 1,8-cineole [49]. Another study indicated that tea tree oil and its major component, terpinen-4-ol, can also interfere with the migration and invasion processes of drug-sensitive and drug-resistant melanoma cells [50]. Despite several papers about the cytotoxic potential of terpinen-4-ol on cancer cells and the obtained results against Jurkat cells, this investigation reported weak/very weak cytotoxicity of terpinen-4-ol against J774A.1 and no cytotoxicity against HeLa cells lines.

Considering all previous results for MTT assay, Jurkat cells were chosen for further evaluation using LDH activity assay and analysis of cell DNA content in order to elucidate the possible mechanism of cytotoxicity.

**Figure 1:**
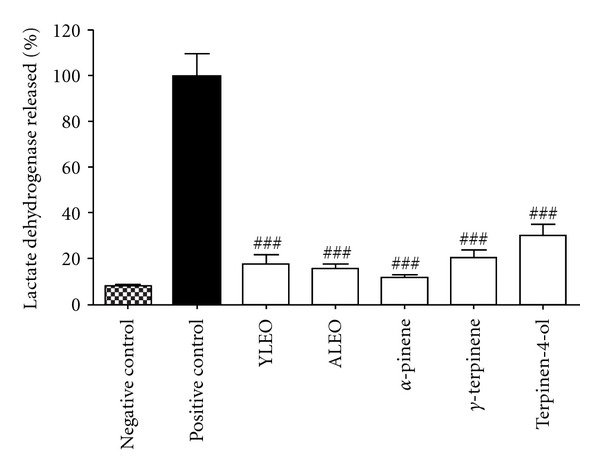
Results of cytoplasmic LDH released of Jurkat cells treated with each sample at 300 *μ*g/mL concentration. Legend: YLEO: young leaves essential oil; ALEO: adult leaves essential oil; positive control: 1% TritonX-100. The results are shown as mean ± SD from three independent experiments. The symbol ^###^ represents a value of *P* < 0.001 that was considered to be highly significant compared to the positive control.

The Figure 1 shows the cytoplasmic LDH released of Jurkat cells treated with each sample at 300 *μ*g/mL concentration. Comparing to serum-free culture medium (negative control), no statistically significant increase in LDH release by Jurkat cells was observed. However, all samples showed a statistically significant difference (*P* < 0.001) in LDH released as compared to 1% TritonX-100 (positive control). Cells in damage or under stress can release cytoplasmic LDH and other substances into the medium due a disruption of cytoplasmic membrane and cell necrosis [19]. Therefore, it is possible to suggest that cytotoxic activity of samples was not based on mechanism of cell death by necrosis. Consequently, these results indicate that cytotoxic activity of samples can involve pathways of inducing cell death by apoptosis.

**Figure 2:**
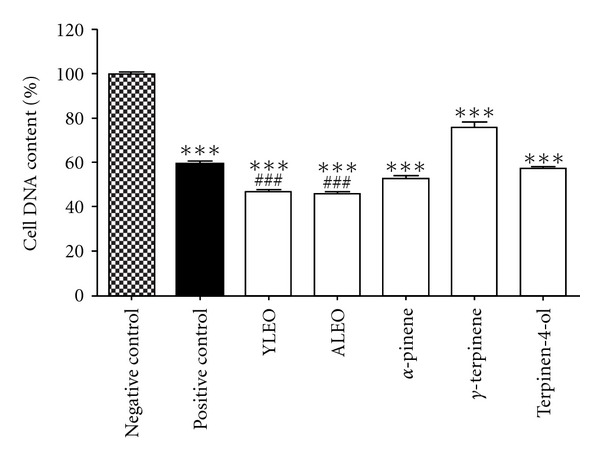
Results of DNA content of Jurkat cells treated with each sample at 300 *μ*g/mL concentration. Legend: YLEO: young leaves essential oil; ALEO: adult leaves essential oil; positive control: vincristine (40 nM). The results are shown as mean ± SD from three independent experiments. The symbols *** and ^###^ represent a value of *P* < 0.001 that was considered to be highly significant compared to the negative and positive controls, respectively.

The effect of samples on proliferation of Jurkat cell by measuring DNA content is indicated in Figure 2. At 300 *μ*g/mL concentration, all samples led to a statistically significant difference (*P* < 0.001) in DNA content as compared to RPMI 1640 containing 10% FBS that was used as negative control. Furthermore, the essential oils from young and adult leaves of *E. benthamii *also demonstrated a statistically significant decrease in DNA content as compared to 40 nM vincristine (positive control). The analysis of cell DNA content revealed that the studied samples, particularly the volatile oil of *E. benthamii*, can inhibit the proliferation of cancer cells probably as a result of cytotoxicity, previously verified by MTT assay.

Considering the few number of investigations about cytotoxic effects of *Eucalyptus* spp. on tumor cells, this paper reported that essential oils from young and adult leaves of *E. benthamii* present cytotoxicity mainly against Jurkat and HeLa cell lines in comparing to the isolated terpenes, particularly **α*-*pinene and **γ**-terpinene. The obtained results also demonstrate the importance of the *E. benthamii* as an alternative herbal source of a complex mixture of volatile compounds that can be used as cytotoxic agent.

 Although the essential oils provided by *Eucalyptus* species have been used in folk medicine, it is important to mention that their use must be cautious because a systemic toxicity can occur from ingestion or topical application at higher doses as widely reported [51–54]. The probable lethal dose of pure essential oil of *Eucalyptus* spp. for an adult is in the range of 0.05 mL to 0.5 mL/kg, and severe poisoning has occurred in children after ingestion of 4 to 5 mL.

## 4. Conclusion

In general, these findings demonstrated that the essential oils of *E. benthamii* show improved cytotoxic potential than the isolated terpenes **α*-*pinene and **γ**-terpinene. Moreover, the obtained results support an experimental basis for reporting that the essential oils of *E. benthamii* lead to cell death mostly by apoptotic process. Furthermore, these data can also pave the way for future development of therapeutic opportunities against cancer.

## Figures and Tables

**Table 1:** Chemical composition of the essential oil from young and adult leaves of *E. benthamii*.

Volatile compound	RT	RI	Peak area (%)		Identification
			YLEO	ALEO	
**α**-pinene	4.90	935	36.82	36.92	RI, MS
**β**-pinene	5.90	977	nd	0.48	RI, MS
*p*-cimene	7.27	1024	1.59	1.90	RI, MS
Limonene	7.39	1028	0.81	0.90	RI, MS
**γ**-terpinene	8.38	1058	5.51	4.38	RI, MS
(*E*)-pinocarveol	11.29	1138	nd	0.84	RI, MS
terpinen-4-ol	12.82	1177	1.23	1.05	RI, MS
**α**-terpineol	13.39	1192	1.32	2.02	RI, MS
**α**-gurjunene	22.25	1406	1.70	1.29	RI, MS
Aromadendrene	23.50	1437	15.94	12.40	RI, MS
*allo*-aromadendrene	24.30	1457	1.98	1.48	RI, MS
Viridiflorene	25.69	1492	1.47	1.13	RI, MS
Globulol	29.25	1584	20.54	20.22	RI, MS
Viridiflorol	29.47	1590	1.63	3.13	RI, MS
Rosifolius	29.84	1599	1.63	1.60	RI, MS
10-*epi*-**γ**-eudesmol	30.59	1620	1.78	1.64	RI, MS
Compounds identified					
Monoterpene hydrocarbons			44.73	44.58	
Oxygenated monoterpenes			2.55	3.91	
Sesquiterpene hydrocarbons			21.09	16.30	
Oxygenated sesquiterpenes			25.58	26.59	

Legend: YLEO: young leaves essential oil; ALEO: adult leaves essential oil; RT: retention time; RI: retention index; MS: mass spectroscopy; nd: not detected.

**Table 2:** Evaluation of cytotoxicity by MTT assay in cell lines after 24 h.

Cell line	IC_50_ (*μ*g/mL)				
	YLEO	ALEO	**α**-pinene	**γ**-terpinene	terpinen-4-ol
Jurkat	108.33 ± 1.83	54.96 ± 5.80	192.42 ± 9.38	136.6 ± 10.67	50.20 ± 13.03
J774A.1	287.98 ± 3.54	252.55 ± 1.91	>300	>300	220.02 ± 7.15
HeLa	84.24 ± 5.94	110.02 ± 2.89	>300	>300	>300

Legend: YLEO: young leaves essential oil; ALEO: adult leaves essential oil; IC_50_: concentration that reduces the mitochondrial activity by 50%. The results are shown as mean ± SD from three independent experiments.

**Table 3:** Evaluation of cytotoxicity by MTT assay in cell lines after 72 h.

Cell line	IC_50_ (*μ*g/mL)				
	YLEO	ALEO	**α**-pinene	**γ**-terpinene	terpinen-4-ol
Jurkat	56.51 ± 1.48	36.63 ± 3.52	186.09 ± 37.98	156.92 ± 10.22	54.84 ± 3.85
J774A.1	166.87 ± 5.77	178.85 ± 6.11	>300	>300	189.7 ± 14.74
HeLa	120.57 ± 6.22	101.90 ± 19.47	>300	>300	>300

Legend: YLEO: young leaves essential oil; ALEO: adult leaves essential oil; IC_50_: concentration that reduces the mitochondrial activity by 50%. The results are shown as mean ± SD from three independent experiments.

## References

[1] Brooker MIH, Kleinig DA (2006). *Field Guide to Eucalyptus*.

[2] Chevallier A (2001). *Encyclopedia of Medicinal Plants*.

[3] Silva J, Abebe W, Sousa SM, Duarte VG, Machado MIL, Matos FJA (2003). Analgesic and anti-inflammatory effects of essential oils of *Eucalyptus*. *Journal of Ethnopharmacology*.

[4] Williams LR, Stockley JK, Yan W, Home VN (1998). Essential oils with high antimicrobial activity for therapeutic use. *International Journal of Aromatherapy*.

[5] Benyahia S, Benayache S, Benayache F (2005). Cladocalol, a pentacyclic 28-nor-triterpene from *Eucalyptus cladocalyx* with cytotoxic activity. *Phytochemistry*.

[6] Takasaki M, Konoshima T, Etoh H, Singh IP, Tokuda H, Nishino H (2000). Cancer chemopreventive activity of euglobal-G1 from leaves of *Eucalyptus grandis*. *Cancer Letters*.

[7] Ito H, Koreishi M, Tokuda H, Nishino H, Yoshida T (2000). Cypellocarpins A-C, phenol glycosides esterified with oleuropeic acid, from *Eucalyptus cypellocarpa*. *Journal of Natural Products*.

[8] Ashour HM (2008). Antibacterial, antifungal, and anticancer activities of volatile oils and extracts from stems, leaves, and flowers of *Eucalyptus sideroxylon* and *Eucalyptus torquata*. *Cancer Biology and Therapy*.

[9] Al-Fatimi M, Friedrich U, Jenett-Siems K (2005). Cytotoxicity of plants used in traditional medicine in Yemen. *Fitoterapia*.

[10] NSW—National Parks & Wildlife Service (2000). *Threatened species information: *Eucalyptus* benthamii Maiden and Cambage*.

[11] Costa EM A madeira do eucalipto na indústria moveleira.

[12] Ebejer WM Uso de plantas por agricultores familiares.

[13] Lucia A, Juan LW, Zerba EN, Harrand L, Marcó M, Masuh HM Validation of models to estimate the fumigant and larvicidal activity of *Eucalyptus* essential oils against *Aedes aegypti* (Diptera: Culicidae).

[14] Adams RP (2007). *Identification of Essential Oil Components by Gas Chromatography/Mass Spectroscopy*.

[15] Virador VM, Kobayashi N, Matsunaga J, Hearing VJ (1999). A standardized protocol for assessing regulators of pigmentation. *Analytical Biochemistry*.

[16] Sylvestre M, Pichette A, Longtin A, Nagau F, Legault J (2006). Essential oil analysis and anticancer activity of leaf essential oil of Croton flavens L. from Guadeloupe. *Journal of Ethnopharmacology*.

[17] Cardile V, Russo A, Formisano C (2009). Essential oils of *Salvia bracteata* and *Salvia rubifolia* from lebanon: chemical composition, antimicrobial activity and inhibitory effect on human melanoma cells. *Journal of Ethnopharmacology*.

[18] Mosmann T (1983). Rapid colorimetric assay for cellular growth and survival: application to proliferation and cytotoxicity assays. *Journal of Immunological Methods*.

[19] Korzeniewski C, Callewaert DM (1983). An enzyme-release assay for natural cytotoxicity. *Journal of Immunological Methods*.

[20] Sellitti DF, Suzuki K, Doi SQ (2001). Thyroglobulin increases cell proliferation and suppresses Pax-8 in mesangial cells. *Biochemical and Biophysical Research Communications*.

[21] Gras EK, Read J, Mach CT, Sanson GD, Clissold FJ (2005). Herbivore damage, resource richness and putative defences in juvenile versus adult *Eucalyptus* leaves. *Australian Journal of Botany*.

[22] Mahmoud SS, Croteau RB (2001). Metabolic engineering of essential oil yield and composition in mint by altering expression of deoxyxylulose phosphate reductoisomerase and menthofuran synthase. *Proceedings of the National Academy of Sciences of the United States of America*.

[23] Tian Y-H, Liu X-M, Zhou Y-H, Qin R-H (2005). Chemical composition of essential oils of leaves from *Eucalyptus camaldulensis* and *Eucalyptus benthamii*. *Jingxi Huagong*.

[24] Silva PHM, Brito JO, Silva FG (2006). Potential of eleven *Eucalyptus* species for the production of essential oils. *Scientia Agricola*.

[25] Mossi AJ, Astolfi V, Kubiak G (2011). Insecticidal and repellency activity of essential oil of *Eucalyptus* sp. against *Sitophilus zeamais* motschulsky (Coleoptera, Curculionidae). *Journal of the Science of Food and Agriculture*.

[26] Elaissi A, Salah KH, Mabrouk S, Larbi KM, Chemli R, Harzallah-Skhiri F (2011). Antibacterial activity and chemical composition of 20 *Eucalyptus* species’ essential oils. *Food Chemistry*.

[27] Gairola S, Gupta V, Bansal P, Singh R, Maithani M (2010). Herbal antitussives and expectorants—a review. *International Journal of Pharmaceutical Sciences Review and Research*.

[28] Bakkali F, Averbeck S, Averbeck D, Idaomar M (2008). Biological effects of essential oils—a review. *Food and Chemical Toxicology*.

[29] Richter C, Schlegel J (1993). Mitochondrial calcium release induced by prooxidants. *Toxicology Letters*.

[30] Novgorodov SA, Gudz TI (1996). Permeability transition pore of the inner mitochondrial membrane can operate in two open states with different selectivities. *Journal of Bioenergetics and Biomembranes*.

[31] Vercesi AE, Kowaltowski AJ, Grijalba MT, Meinicke AR, Castilho RF (1997). The role of reactive oxygen species in mitochondrial permeability transition. *Bioscience Reports*.

[32] De Sousa AC, Alviano DS, Blank AF, Alves PB, Alviano CS, Gattas CR (2004). *Melissa officinalis* L. Essential oil: antitumoral and antioxidant activities. *Journal of Agricultural and Food Chemistry*.

[33] Leite A, Lima E, Souza E, Diniz M, Trajano V, Medeiros I (2007). Inhibitory effect of *β*-pinene, *α*-pinene and eugenol on the growth of potential infectious endocarditis causing Gram-positive bacteria. *Revista Brasileira de Ciencias Farmaceuticas*.

[34] Merck CD (2001). *The Merck Index: an Encyclopedia of Chemicals, Drugs and Biologycals*.

[35] Setzer WN, Setzer MC, Moriarity DM, Bates RB, Haber WA (1999). Biological activity of the essential oil of *Myrcianthes* sp. nov. black fruit from monteverde, costa rica. *Planta Medica*.

[36] Dar MY, Shah WA, Rather MA, Qurishi Y, Hamid A, Qurishi MA (2011). Chemical composition, in vitro cytotoxic and antioxidant activities of the essential oil and major constituents of Cymbopogon jawarancusa (Kashmir). *Food Chemistry*.

[37] Loizzo MR, Tundis R, Menichini F, Saab AM, Statti GA, Menichini F (2008). Antiproliferative effects of essential oils and their major constituents in human renal adenocarcinoma and amelanotic melanoma cells. *Cell Proliferation*.

[38] Zhou JY, Tang FD, Mao GG, Bian RL (2004). Effect of *α*-*pinene* on nuclear translocation of nf-*κ*b in THP-1 cells. *Acta Pharmacologica Sinica*.

[39] Romeilah RM (2009). Anticancer and antioxidant activities of *Matricaria chamomilla* L. and *Marjorana hortensis* essential oils. *Research Journal of Medicine and Medical Sciences*.

[40] Bourgou S, Pichette A, Marzouk B, Legault J (2010). Bioactivities of black cumin essential oil and its main terpenes from Tunisia. *South African Journal of Botany*.

[41] Astani A, Reichling J, Schnitzler P (2010). Comparative study on the antiviral activity of selected monoterpenes derived from essential oils. *Phytotherapy Research*.

[42] Cha JD, Jeong MR, Jeong SI (2007). Chemical composition and antimicrobial activity of the essential oil of *Cryptomeria japonica*. *Phytotherapy Research*.

[43] Barra A, Coroneo V, Dessi S, Cabras P, Angioni A (2007). Characterization of the volatile constituents in the essential oil of *Pistacia lentiscus* L. from different origins and its antifungal and antioxidant activity. *Journal of Agricultural and Food Chemistry*.

[44] Wedge DE, Tabanca N, Sampson BJ (2009). Antifungal and insecticidal activity of two *juniperus* essential oils. *Natural Product Communications*.

[45] Zúñiga B, Guevara-Fefer P, Herrera J (2005). Chemical composition and anti-inflammatory activity of the volatile fractions from the bark of eight mexican *Bursera* species. *Planta Medica*.

[46] Greay SJ, Ireland DJ, Kissick HT (2010). Induction of necrosis and cell cycle arrest in murine cancer cell lines by *Melaleuca alternifolia* (tea tree) oil and terpinen-4-ol. *Cancer Chemotherapy and Pharmacology*.

[47] Calcabrini A, Stringaro A, Toccacieli L (2004). Terpinen-4-ol, the main component of *Melaleuca alternifolia* (tea tree) oil inhibits the in vitro growth of human melanoma cells. *The Journal of Investigative Dermatology*.

[48] Wu C-S, Chen Y-J, Chen JJW (2012). Terpinen-4-ol induces apoptosis in human nonsmall cell lung cancer in vitro and in vivo. *Evidence-Based Complementary and Alternative Medicine*.

[49] Hayes AJ, Leach DN, Markham JL, Markovic B (1997). *In vitro* cytotoxicity of Australian tea tree oil using human cell lines. *Journal of Essential Oil Research*.

[50] Bozzuto G, Colone M, Toccacieli L, Stringaro A, Molinari A (2011). Tea tree oil might combat melanoma. *Planta Medica*.

[51] Allan J (1910). Poisoning by oil of *Eucalyptus*. *British Medical Journal*.

[52] Foggie WE (2011). Eucalyptus oil poisoning. *British Medical Journal*.

[53] Hindle RC (1994). *Eucalyptus* oil ingestion. *New Zealand Medical Journal*.

[54] Darben T, Cominos B, Lee CT (1998). Topical *Eucalyptus* oil poisoning. *Australasian Journal of Dermatology*.

